# Adeno-associated virus serotype rh.10 displays strong muscle tropism following intraperitoneal delivery

**DOI:** 10.1038/srep40336

**Published:** 2017-01-09

**Authors:** Jianzhong Ai, Jia Li, Dominic J. Gessler, Qin Su, Qiang Wei, Hong Li, Guangping Gao

**Affiliations:** 1 Institute of Urology, Department of Urology, West China Hospital, Sichuan University, Chengdu, Sichuan, P.R. China; 2 Horae Gene Therapy Center, University of Massachusetts Medical School, Worcester, Massachusetts, USA; 3 Department of Microbiology and Physiology Systems, University of Massachusetts Medical School, Worcester, Massachusetts, USA; 4 State Key Laboratory of Biotherapy, West China Hospital, Sichuan University, Chengdu, P.R. China

## Abstract

Recombinant adeno-associated virus (rAAV) is an attractive tool for basic science and translational medicine including gene therapy, due to the versatility in its cell and organ transduction. Previous work indicates that rAAV transduction patterns are highly dependent on route of administration. Based on this relationship, we hypothesized that intraperitoneal (IP) administration of rAAV produces unique patterns of tissue tropism. To test this hypothesis, we investigated the transduction efficiency of 12 rAAV serotypes carrying an enhanced green fluorescent protein (EGFP) reporter gene in a panel of 12 organs after IP injection. Our data suggest that IP administration emphasizes transduction patterns that are different from previously reported intravascular delivery methods. Using this approach, rAAV efficiently transduces the liver, pancreas, skeletal muscle, heart and diaphragm without causing significant histopathological changes. Of note, rAAVrh.10 showed excellent muscle transduction following IP administration, highlighting its potential as a new muscle-targeting vector.

Over the last decade, recombinant adeno-associated virus (rAAV) has been developed into a powerful gene delivery tool for use in both basic research applications and in clinical trials^1^,^2^. Compared to other viruses, rAAV possesses many advantages for gene delivery, including low immunogenicity and genotoxicity, long-term gene expression, wide tissue tropism and high transduction efficiency *in vivo*^3^,^4^.

rAAV can efficiently transduce many organs, including liver, heart, eyes and muscle, and its tissue transduction preferences are dependent both on route of administration and the properties of a given AAV capsid^5^. Several groups have shown that route of administration is a secondary determinant of AAV tropism. For example, after intraperitoneal (IP) injection, rAAV8 transduces skeletal muscle and heart efficiently, whereas it primarily transduces the liver following intravenous (IV) administration^6^,^7^. Interestingly, Guo *et al*. demonstrated that intrathecal injection of rAAVrh.10 into the lumbar cistern leads to transgene expression in 60 to 90% of the cells in the spinal cord^8^. Overall, these studies indicate that rAAV transduces specific and varying organs following systemic or local injection. IP injection is less invasive than many types of local injection and potentially induces less of a humoral immune response in comparison to other routes of delivery, such as IV administration^9^,^10^,^11^,^12^. In addition, recent studies have shown that IP administration of certain rAAV serotypes produces comparable transduction efficiency relative to other routes of administration^9^,^13^,^14^.

In addition to route of administration, the properties of the AAV capsid act as the primary determinant of tissue tropism. This is advantageous, as a plethora of AAV capsids that display different transduction patterns have been discovered in recent decades. Out of those capsids, at least 12 serotypes of rAAV are commonly used for basic research and clinical studies: rAAV2, rAAV3b, rAAV5, rAAV6, rAAV6.2, rAAV7, rAAV8, rAAV9, rAAV rhesus (rh.) 8, rAAVrh.10, rAAVrh.39 and rAAVrh.43^15^. A comprehensive comparison of the transduction patterns for these serotypes following IP administration has not yet been reported. In this study, we performed a side-by-side comparison of all 12 serotypes following IP injection under consistent conditions. This enabled the identification of safe and appropriate rAAV serotypes for future applications in basic and clinical research involving IP administration.

This study represents the first reported investigation into the tropism patterns exhibited by 12 commonly used AAV serotypes following IP injection. To accomplish this, different rAAV capsids carrying an enhanced green fluorescent protein (EGFP) reporter gene were evaluated for transduction efficiency, and their safety profiles were compared. These findings create new avenues for the clinical development of rAAV-based gene therapy vectors and provide new strategies for the treatment of diseases associated with the organs identified as primary targets of certain AAV capsids.

## Results

### rAAV robustly transduces the liver, pancreas, skeletal muscle, heart and diaphragm following IP delivery

Overall, rAAV serotypes 2, 3b, 5, 6 and 6.2 consistently showed weak EGFP expression and low vector genome copies per cell in all 12 organs tested (liver, pancreas, skeletal muscle, heart, diaphragm, lungs, kidneys, spleen, stomach, intestine, bladder and brain; Suppl. Fig. 1–12).

**Figure 1:**
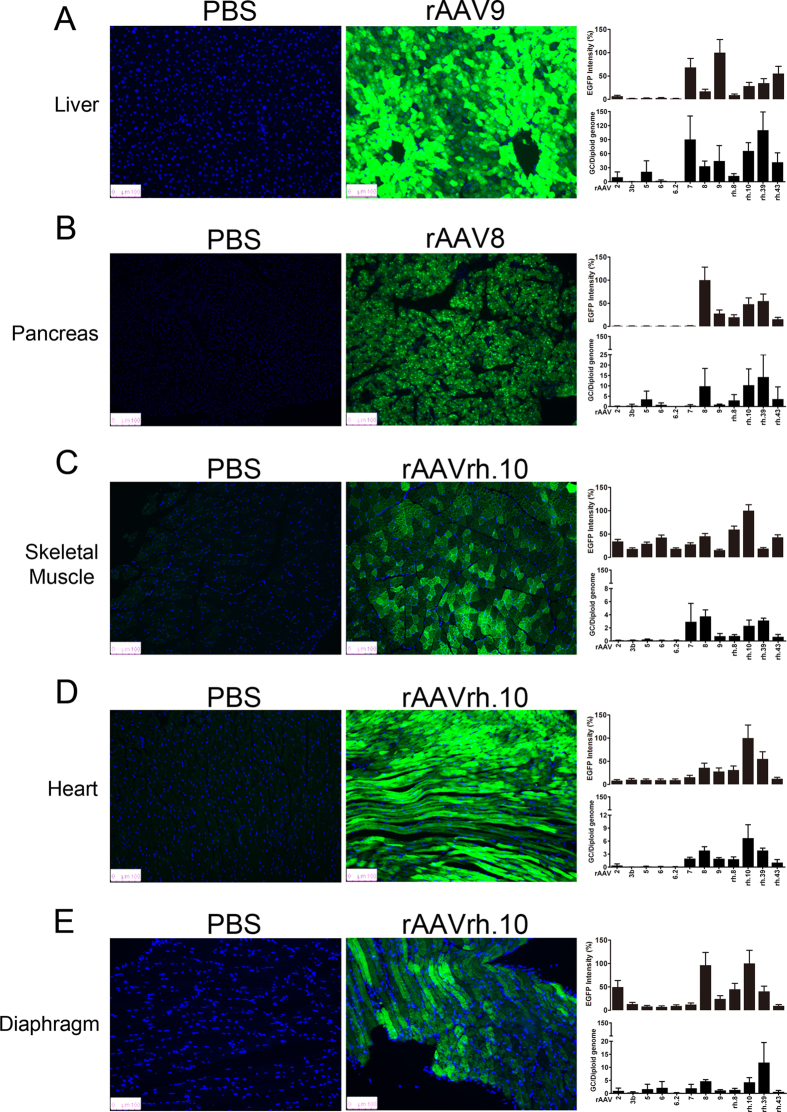
rAAV efficiently transduces mouse liver, pancreas, skeletal muscle, heart and diaphragm. Fluorescence images, EGFP intensity and vector genome copy number quantification for the liver (**A**), pancreas (**B**), skeletal muscle (**C**), heart (**D**), and diaphragm (**E**) of PBS- and rAAV-treated mice.

In contrast to previous publications examining IV rAAV administration, rAAV9 and rAAV7 showed the strongest EGFP expression in the liver; surprisingly, rAAV8 transduced this organ at weaker levels than either of these serotypes, suggesting that IP administration influences tropism^16^. In fact, rAAV8 was one of the weakest transducers of the liver among all serotypes tested based on EGFP intensity (Fig. 1A, Suppl. Fig. 1). In contrast, rAAV8 exhibited the strongest transduction of the pancreas, producing approximately 30% higher EGFP intensity over the next strongest serotype, rAAVrh.39. These results suggest that IP-delivered rAAV8 may be an attractive choice for the development of pancreas-targeted gene delivery vectors, which might be useful for the treatment of diseases such as type I diabetes (Fig. 1B, Suppl. Fig. 2). The performance of rAAV8 in the pancreas was consistent with previous publications^17^.

Next, we analyzed skeletal muscle transduction. rAAVrh.10 showed the most robust transduction of skeletal muscle, followed closely by rAAVrh.8 and rAAV8 (Fig. 1C, Suppl. Fig. 3). rAAVrh.10 also displayed the strongest EGFP expression in the heart and diaphragm (Fig. 1D and E, Suppl. Fig. 4 and 5), suggesting that rAAVrh.10 shows excellent muscle targeting following IP administration. rAAVrh.10 showed relatively weak transduction of other tissues, such as the brain (Suppl. Fig. 11), which seems to be in contrast to the transduction patterns seen for this serotype following IV administration^18^.

### rAAV shows an excellent safety profile

One concern associated with virus-mediated gene delivery is safety, particularly with regard to cell-mediated immune responses. Viral vectors might induce liver-directed immune responses, resulting in cell-mediated liver damage. However, it can also affect the entire organism in a more generalized manner.

**Figure 2:**
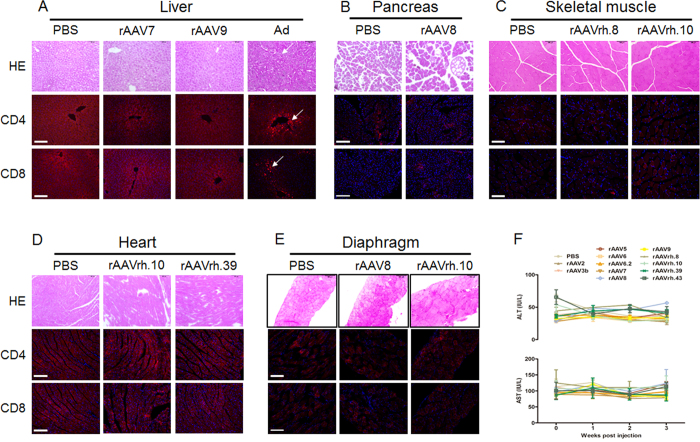
Safety profile of rAAV following IP injection. HE, CD4 and CD8 staining of a mouse liver (**A**), pancreas (**B**), skeletal muscle (**C**), heart (**D**), and diaphragm (**E**). Serum transaminases for all 12 rAAV serotypes (**F**). Ad: adenovirus. White arrow indicate the positive staining of immune cell infiltration.

The surrogate markers alanine aminotransferase (ALT) and aspartate aminotransferase (AST) are released from damaged liver cells predominantly due to induction of T cell responses^19^. Therefore, we measured ALT and AST levels in the sera of the mice treated with the different rAAV serotypes. None of the rAAV serotypes characterized at the vector dose tested in this study induced any significant changes in transaminase levels (Fig. 2F). As this initial screening is only indicative of liver toxicity, regardless of the causative factor, we next applied a second, more detailed method to evaluate safety. Specifically, tissue samples from several organs, including the heart, diaphragm, skeletal muscle, pancreas and liver, were stained to visualize CD4+ and CD8+ cells to determine whether rAAV transduction following IP injection is associated with immune cell infiltration. Similar to PBS-treated mice, no CD4+ or CD8+ cells were detected (Fig. 2A–E) in mice receiving rAAV, suggesting that IP administration of rAAV has an excellent safety profile.

## Discussion

Viral-mediated gene transfer has received intense focus from both basic research and translational medicine researchers^20^. Among the viral vectors developed to date, rAAV has some of the most attractive features for gene delivery, including low immunogenicity, long-term transgene expression, and low genotoxicity^3^,^4^. In recent decades, dozens of AAV serotypes were identified, and some have been developed as viral vectors for efficient gene delivery in different organs. The serotypes showing promise as viral vectors include rAAV2, rAAV3b, rAAV5, rAAV6, rAAV6.2, rAAV7, rAAV8, rAAV9, rAAVrh.8, rAAVrh.10, rAAVrh.39 and rAAVrh.43^15^. These serotypes show varying patterns of transduction efficiency and tissue tropism. For example, rAAV8 and rAAV9 efficiently transduce the pancreas and liver following IV injection^5^,^6^, while rAAV6.2 and rAAV7 show robust transduction of mouse prostate cells *in vivo* following intraprostatic injection^15^.

Compared with other routes of delivery, IP administration offers several advantages, including technical simplicity, minimal induction of humoral immune response and the ability to achieve long-term transgene expression^9^,^11^,^12^. Lei Xu *et al*. utilized IP-injected viral vectors to deliver FKRP gene therapy to restore functional glycosylation of α-dystroglycan and improve muscle function in FKRP-related muscular dystrophy^21^. In another pre-clinical trial, Kok *et al*. rescued the neonatal lethality of argininosuccinate synthetase-deficient mice^14^. In the clinic, a modified adenovirus vector with deletion of the *E1B* 55-kd gene was delivered via IP administration to treat patients with ovarian cancer^22^.

Interestingly, Wang *et al*. evaluated rAAV transduction efficiency following IP injection of rAAV 1, 2, 5, 6, 7 and 8 and found that rAAV8 exhibited the most robust transduction of heart and skeletal muscle^6^. However, our findings suggest that rAAVrh.10 is superior to rAAV8 in transducing the heart, diaphragm and skeletal muscle. In fact, in our hands, rAAV8 ranked third in transducing muscle, suggesting that rAAVrh.10 might be a new leading rAAV candidate for targeting muscle in patients with muscle-related disorders.

In addition to serotype, route of administration is an important factor in determining transduction characteristics. Zincarelli *et al*. evaluated the transduction efficiency and tropism in mice of rAAV serotypes 1–9 based on luciferase reporter gene expression following IV injection and found rAAV9 to be the leading vector for muscle transduction^23^. Although the conditions differed from our study, we showed that rh.10 might be the new leading vector for muscle transduction for applications utilizing IP administration, as it outperformed rAAV9 in this context. Moreover, Gao *et al*. reported that the transduction efficiency of rAAV8 was significantly higher than that of rAAV7 following intraportal injection^24^. Again, the conditions differed from our study. However, our study showed that rAAV7 transduced the liver more efficiently than rAAV8 following IP injection (Fig. 1 and Suppl. Fig. 1). Collectively, these findings suggest that the transduction efficiency and tropism of rAAV in the liver are influenced by the route of administration.

We also obtained fluorescent images of a collection of organs with equal exposure times across all serotypes to avoid biases. Some serotypes did not show an obvious EGFP signal in our images under this condition, even though these organs were indeed transduced with rAAV vectors: the liver was transduced by rAAV8, skeletal muscle was transduced by rAAV9, and the pancreas was transduced by rAAV7.

The results obtained following IP administration of the leading rAAV serotypes confirm the superiority of rAAV8 in transducing the pancreas (Fig. 1B and Suppl. Fig. 2)^17^. Most importantly, our study shows that, following IP administration, rAAVrh.10 is the most efficient serotype at targeting muscle tissue, including skeletal muscle, the heart and the diaphragm (Fig. 1C,D,E and Suppl. Fig. 3, 4 and 5). These findings suggest that rAAVrh.10 is a lead candidate vector for applications involving these tissues. Indeed, the propensity of rh.10 to transduce muscle is highly relevant to muscle-related disorders. It allows for muscle targeting with high transduction efficiency without the need for multiple intramuscular injections, which in the case of the diaphragm would be highly invasive; it also avoids the need for intravascular injection to deliver rAAV, which results in the simultaneous targeting of various undesired organs, such as the brain, liver and intestine (Suppl. Fig. 1, 9 and 11)^18^,^25^. Nevertheless, application-specific studies are needed to determine the optimal doses of rAAV needed for different targeted tissues to treat disorders such as Duchenne muscular dystrophy. Furthermore, to evaluate the translatability of this approach, large animal studies are warranted. The results from such studies will provide further guidance for the selection of rAAV serotypes with specific tissue targeting patterns for use *in vivo*.

In the present study, we administered rAAV at a dose of 1 × 10^12^ GC via IP injection. This dose is equivalent to 4.35 × 10^13^ GC/kg based on the average weight of an 8-week-old mouse (23 g). Although this dose appears to be high, a preclinical study in dogs using semi-systemic intraportal administration of rAAV8 reported the safe administration of 4.95 × 10^13^ GC/kg, supporting the safety and feasibility of our dose^26^. Interestingly, Chuhong Hu *et al*. reported that a lower dose of rAAVrh.10 (2 × 10^10^ per neonatal mouse) produced strong skeletal muscle transduction; however, this study utilized IV administration and examined mice at a younger age than those used here^27^.

In summary, our study demonstrates that IP administration of rAAV offers a semi-systemic route of administration that creates unique tropism patterns for rAAV wherein transduction is robust in certain targeted tissues and simultaneously limited in others that are not of therapeutic importance. In addition, IP administration of rAAV results in an excellent safety profile with no detectable cellular immune response. Finally, we showed that rAAVrh.10 is superior to all previously reported vectors for skeletal, diaphragm and heart muscle transduction when administered through IP injection.

## Materials and Methods

### Production of rAAV vectors

rAAV vectors were produced using the standard triple-transfection method as described previously^28^. The *cis* plasmid used for production encoded an EGFP expression cassette driven by the ubiquitously expressed chicken β-actin (CBA) promoter.

### AAV titration

Viruses were purified by ultracentrifugation over a cesium chloride (CsCl) gradient. Purified viruses were titrated both by quantitative polymerase chain reaction (qPCR) and silver staining. For silver staining, the capsid proteins of purified viruses were compared against standard capsid samples to quantitate protein-staining density. For qPCR, an EGFP-carrying plasmid was diluted to create a standard curve, and viruses were titrated using Ct values. For each serotype, two different viral preparations were tested.

### Animal studies

Eight-week-old C57BL/6 mice were obtained by in-house breeding. A total of 1 × 10^12^ genome copies (GC) diluted in 100 μL phosphate-buffered saline (PBS) or an equal volume of PBS without viral particles was delivered through IP injection (n = 4). All experimental methods were carried out in accordance with the relevant guidelines. All animal study protocols were approved by the University of Massachusetts Medical School Institutional Animal Care and Use Committee.

### EGFP signal analysis

Mouse organs were harvested 3 weeks after IP injection and fixed in 10% buffered formalin overnight at 4 °C. They were then sequentially dehydrated in 10%, 20% and 30% sucrose overnight at 4 °C. The samples were embedded in optimal cutting temperature (O.C.T.) compound (Sakura Finetek, Torrance, CA, USA) and stored at −80 °C. Next, 8-μm-thick cryo-sections were mounted with buffer containing DAPI, and EGFP signals were observed under a fluorescence microscope. EGFP intensities were obtained using Image J software and normalized against DAPI intensities. The highest EGFP intensity of each organ was set as 100%.

### rAAV biodistribution assay

Tissues were harvested at room temperature (RT), flash frozen in liquid nitrogen, and stored at −80 °C. Total DNA was extracted using a QIAamp DNA Mini Kit (Qiagen, Hilden, Germany) according to the manufacturer’s instructions. In total, 100 nanograms (ng) of total DNA was subjected to TaqMan qPCR targeting the gene encoding EGFP. rAAV genome copies were calculated by comparison against a standard curve generated using linearized plasmid encoding EGFP and normalized to cell numbers based on the assumption that each cell contains 2.75 picograms (pg) of total DNA.

### Histological analysis

Tissues were fixed in 10% buffered formalin overnight at RT, embedded in paraffin and sectioned to 4-micron thickness. The sections were stained with hematoxylin and eosin (HE) and imaged using a bright field microscope (Leica, Buffalo Grove, IL, USA).

### Immunofluorescence staining

Mouse tissues were fixed in 10% buffered formalin overnight at 4 °C and then sequentially dehydrated in 10%, 20% and 30% sucrose overnight at 4 °C. The samples were embedded in O.C.T. compound (Sakura Finetek, Torrance CA, USA) and stored at −80 °C. Eight-micron-thick cryo-sections were permeabilized and blocked with 5% bovine serum albumin (BSA) and 1% Triton X-100 in 1× PBS for two hours at 37 °C. The sections were incubated with primary antibodies against CD4 (1:100 diluted, cat #14-0041-82, eBioscience, San Diego, CA, USA) and CD8a (1:100 diluted, cat #14-0081-82, eBioscience, San Diego, CA, USA) overnight at 4 °C and further incubated with secondary antibodies (Life Technologies) for 1 hour at RT in dark. Finally, the sections were mounted with VECTASHIELD mounting medium containing DAPI (Vector Laboratories, Burlingame, CA, USA).

### Serum ALT and AST assays

Blood was collected by facial vein bleeding before and at 1, 2 and 3 weeks after intraperitoneal injection, and serum was separated using a Microtainer tube with serum separator (cat #365967) from BD (Franklin Lakes, NJ, USA). Alanine aminotransferase (ALT) and aspartate aminotransferase (AST) levels were analyzed using an ALT colorimetric endpoint kit (cat #A526) and an AST colorimetric endpoint kit (cat #A561) from TECO Diagnostics (Anaheim, CA, USA), respectively, per the manufacturers’ instructions.

## Additional Information

**How to cite this article:** Ai, J. *et al*. Adeno-associated virus serotype rh.10 displays strong muscle tropism following intraperitoneal delivery. *Sci. Rep.* **7**, 40336; doi: 10.1038/srep40336 (2017).

**Publisher's note:** Springer Nature remains neutral with regard to jurisdictional claims in published maps and institutional affiliations.

## Supplementary Material

Supplementary Figures and Legends Tables
