# Supplementary material for: Adeno-associated virus serotype rh.10 displays strong muscle tropism following intraperitoneal delivery
Source: Sci Rep. 2017 Jan 9;7:40336. doi: 10.1038/srep40336 (PMC5220346; doi:10.1038/srep40336)
Supplement: Supplementary Figures and Legends Tables [file srep40336-s1.pdf]

Adeno-associated virus serotype rh.10 displays strong muscle tropism following intraperitoneal delivery

Jianzhong Ai [a, b, c], Jia Li [b], Dominic Gessler [b, c], Qin Su [b], Qiang Wei [a], Hong Li [a, *] and Guangping Gao [b, c, d, *]

a. Institute of Urology, Department of Urology, West China Hospital, Sichuan University, Chengdu, Sichuan, P.R. China

b. Horae Gene Therapy Center, University of Massachusetts Medical School, Worcester, Massachusetts, USA

c. Department of Microbiology and Physiology Systems, University of Massachusetts Medical School, Worcester, Massachusetts, USA

d. State Key Laboratory of Biotherapy, West China Hospital, Sichuan University, Chengdu, P.R. China

*. Co-corresponding authors

Send correspondence to:

Guangping Gao, Ph.D.

Horae Gene Therapy Center, University of Massachusetts Medical School

368 Plantation Street, AS6-2049

Worcester, MA 01605 USA

Telephone: +1-508-856-3563

FAX: +1-508-856-1552

E-mail: guangping.gao@umassmed.edu

or:

Hong Li, M.D.

Department of Urology, West China Hospital, Sichuan University

Guoxue Xiang # 37

Chengdu, Sichuan 610041 P.R. China

Telephone: +86-028-85125449

FAX: +86-028-85422444

E-mail: lihong_19560707@163.com

Supplementary Figure 1

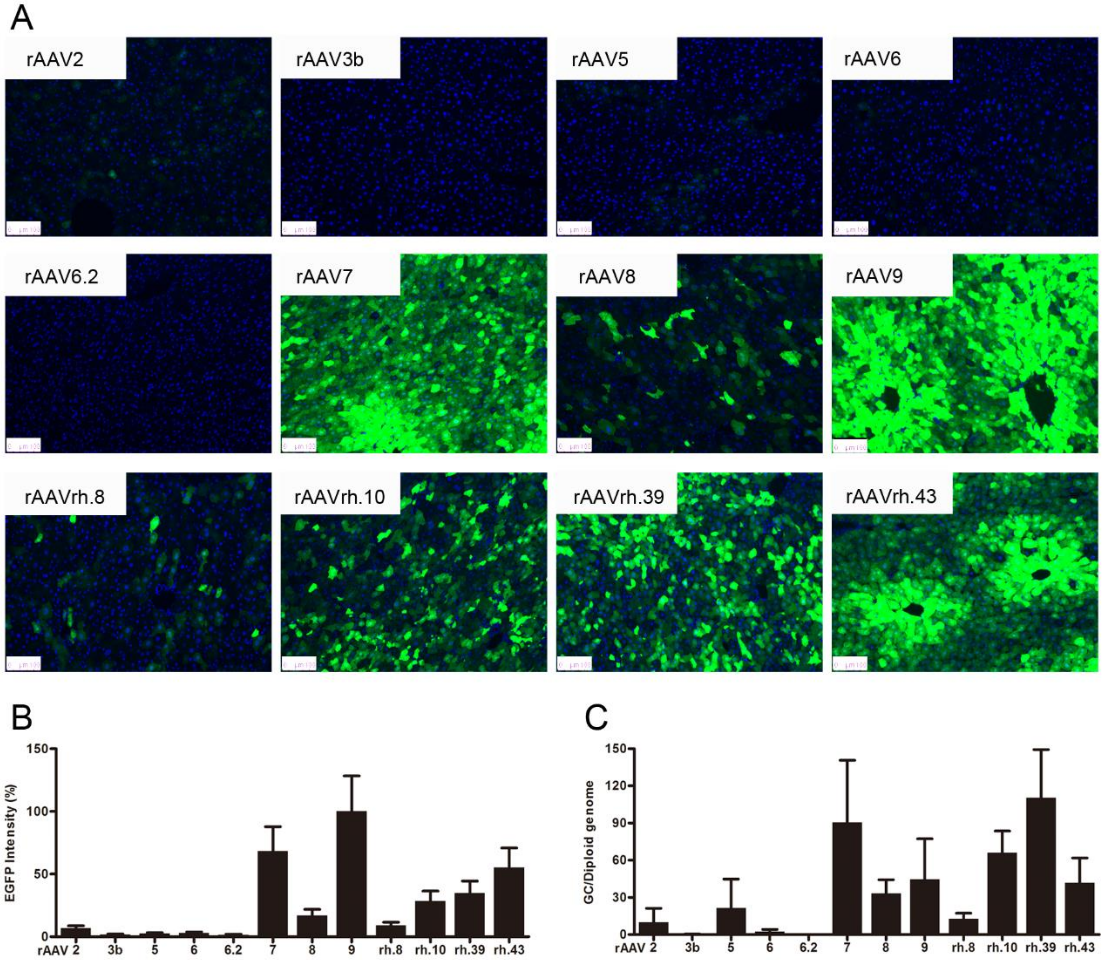

Supplementary Figure 2

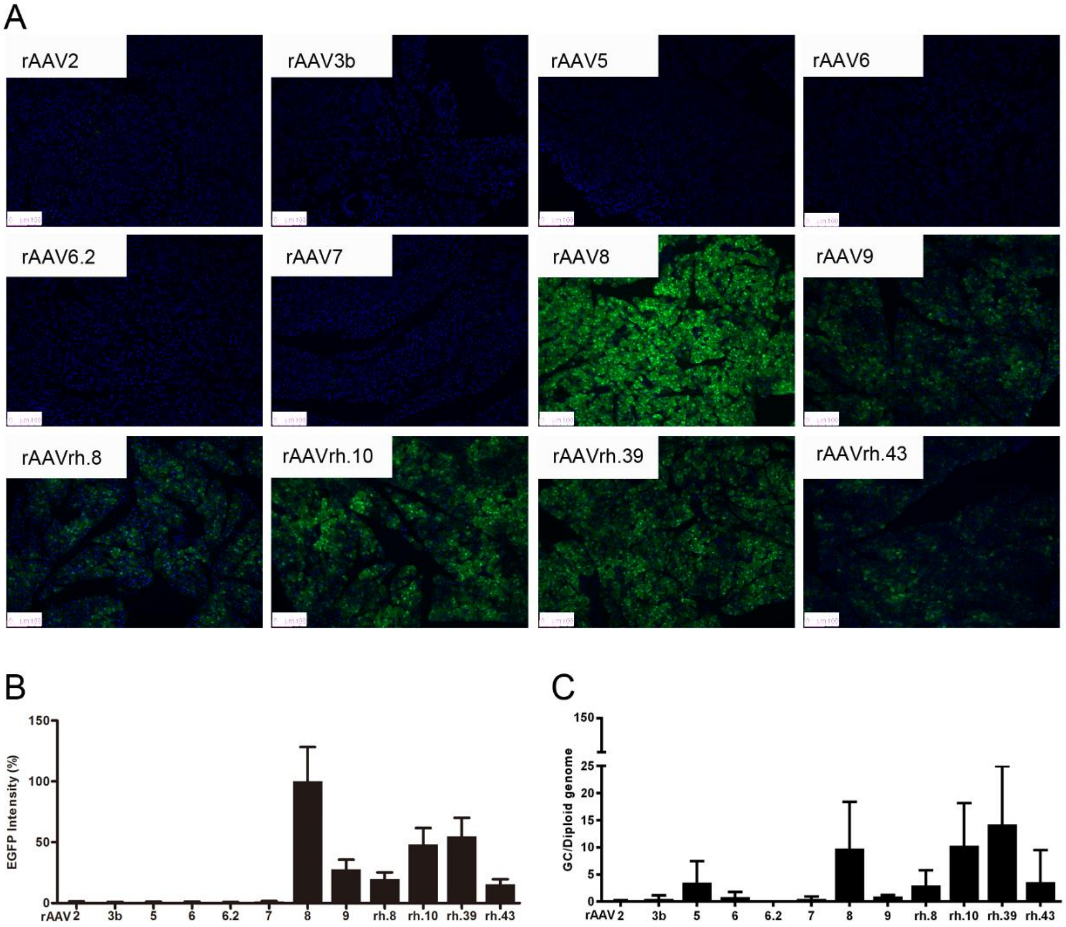

Supplementary Figure 3

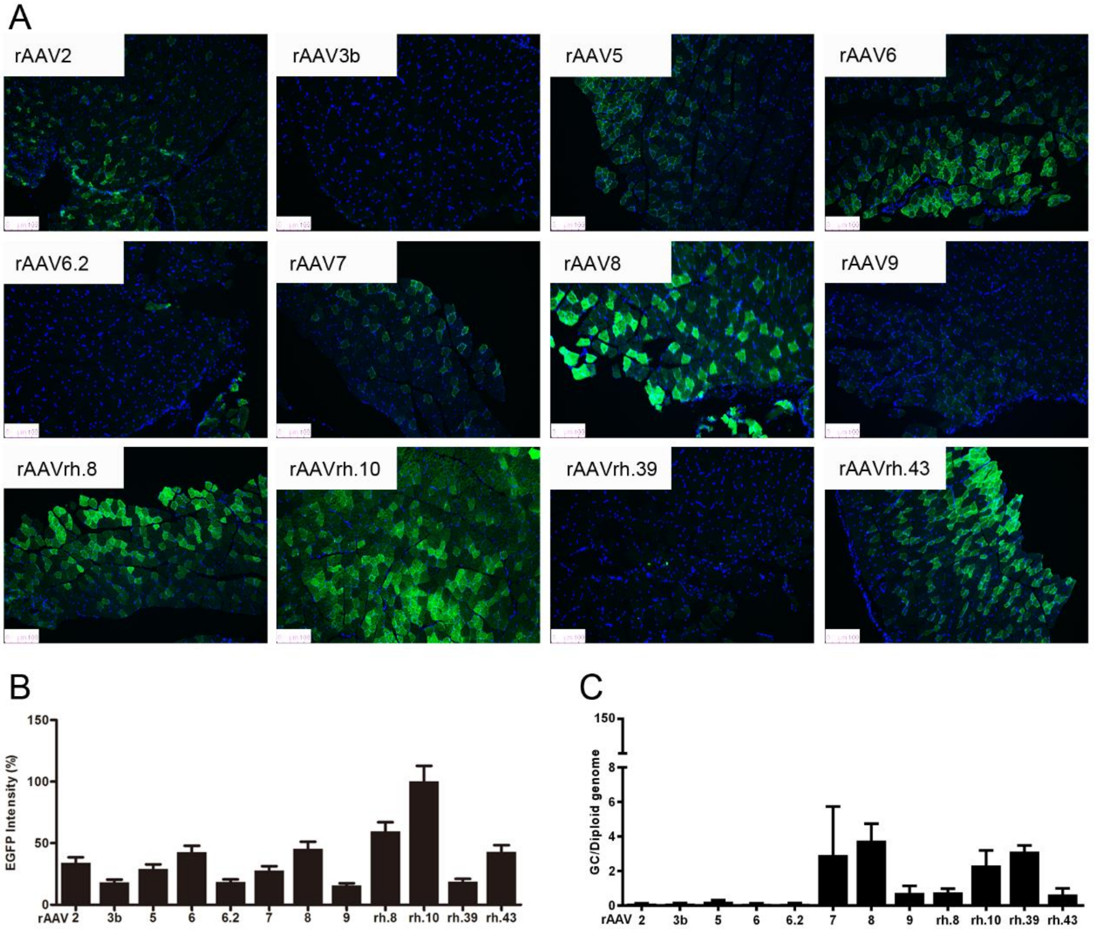

Supplementary Figure 4

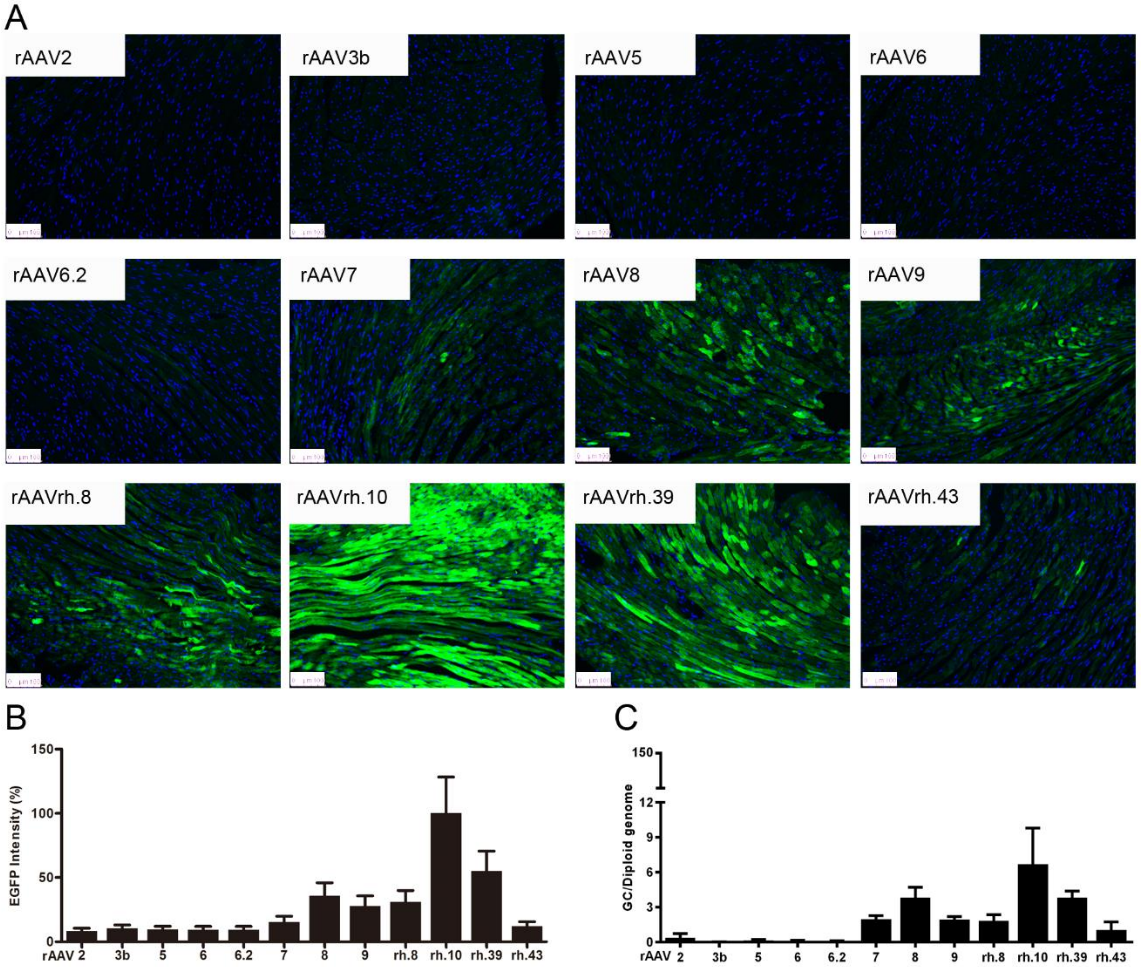

Supplementary Figure 5

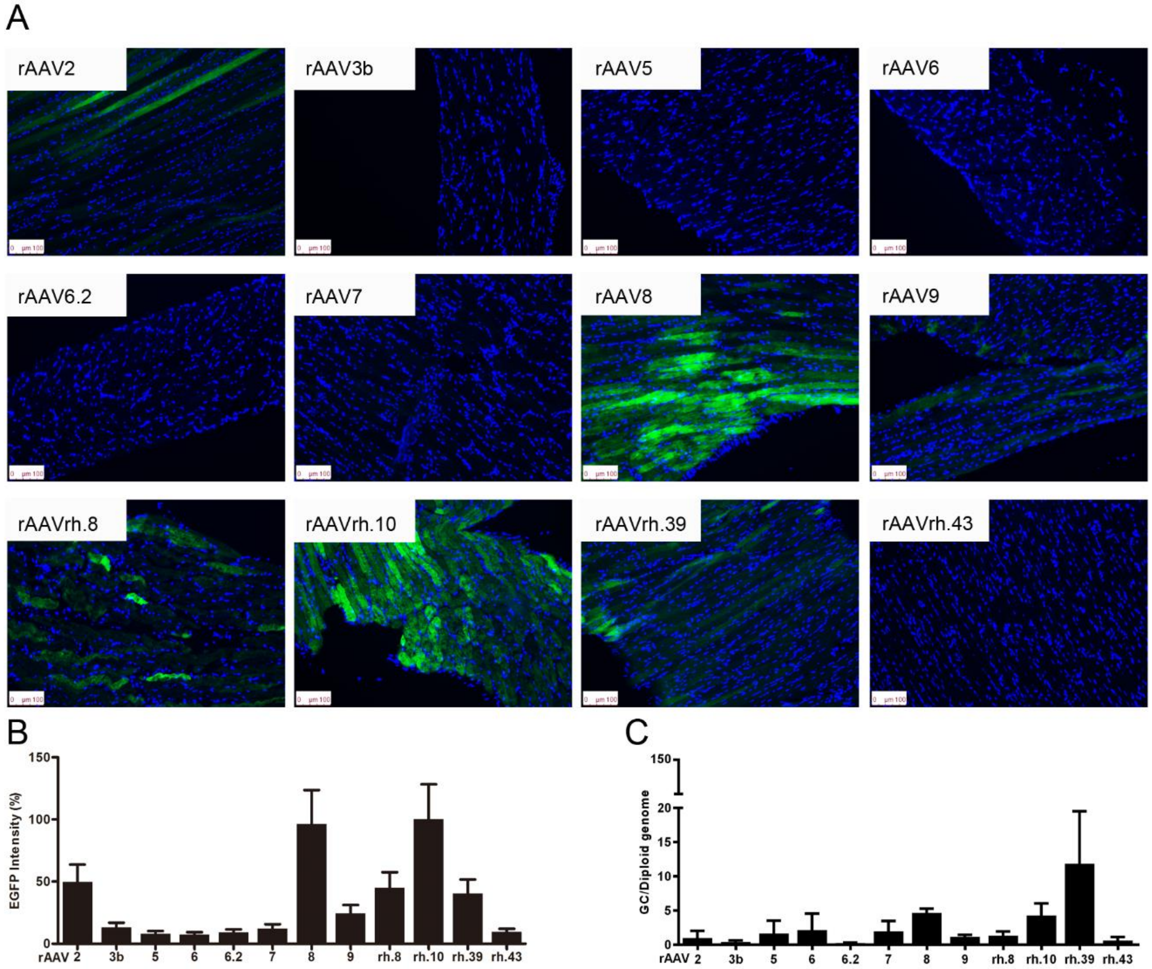

Supplementary Figure 6

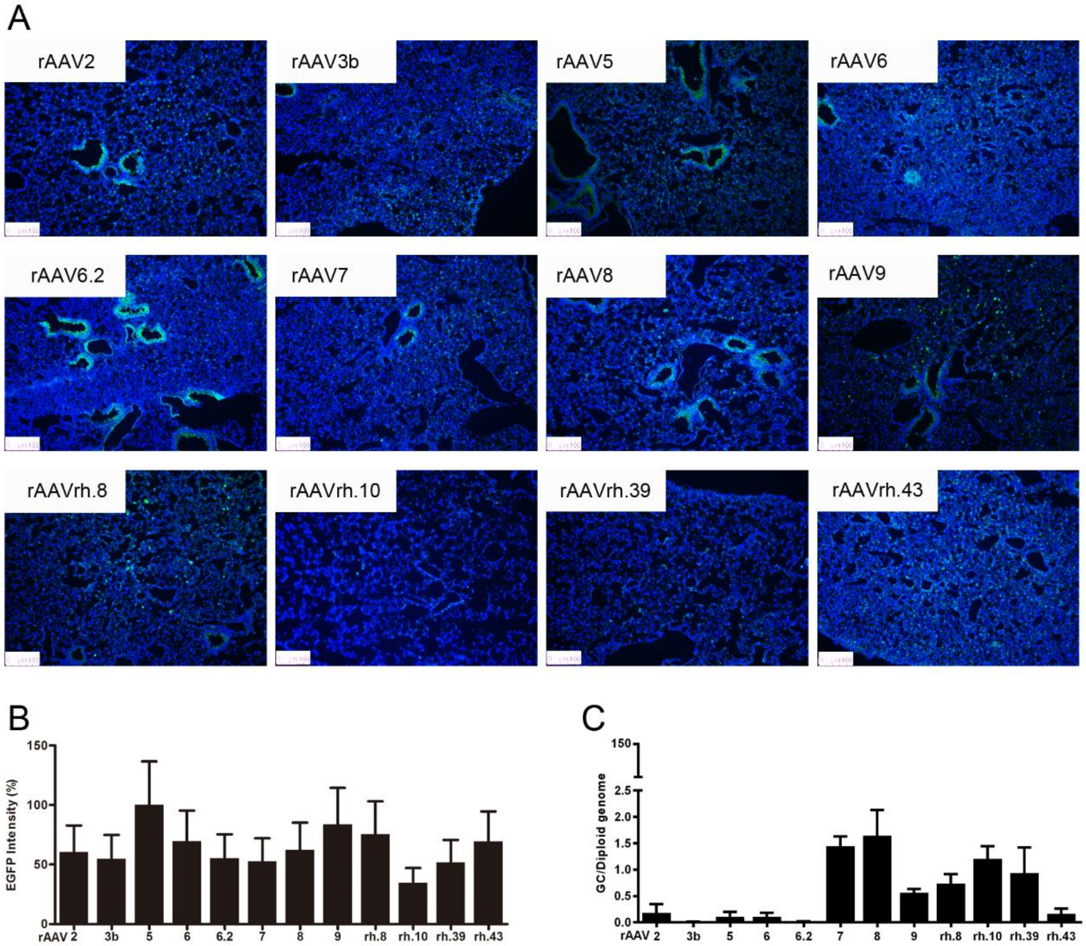

Supplementary Figure 7

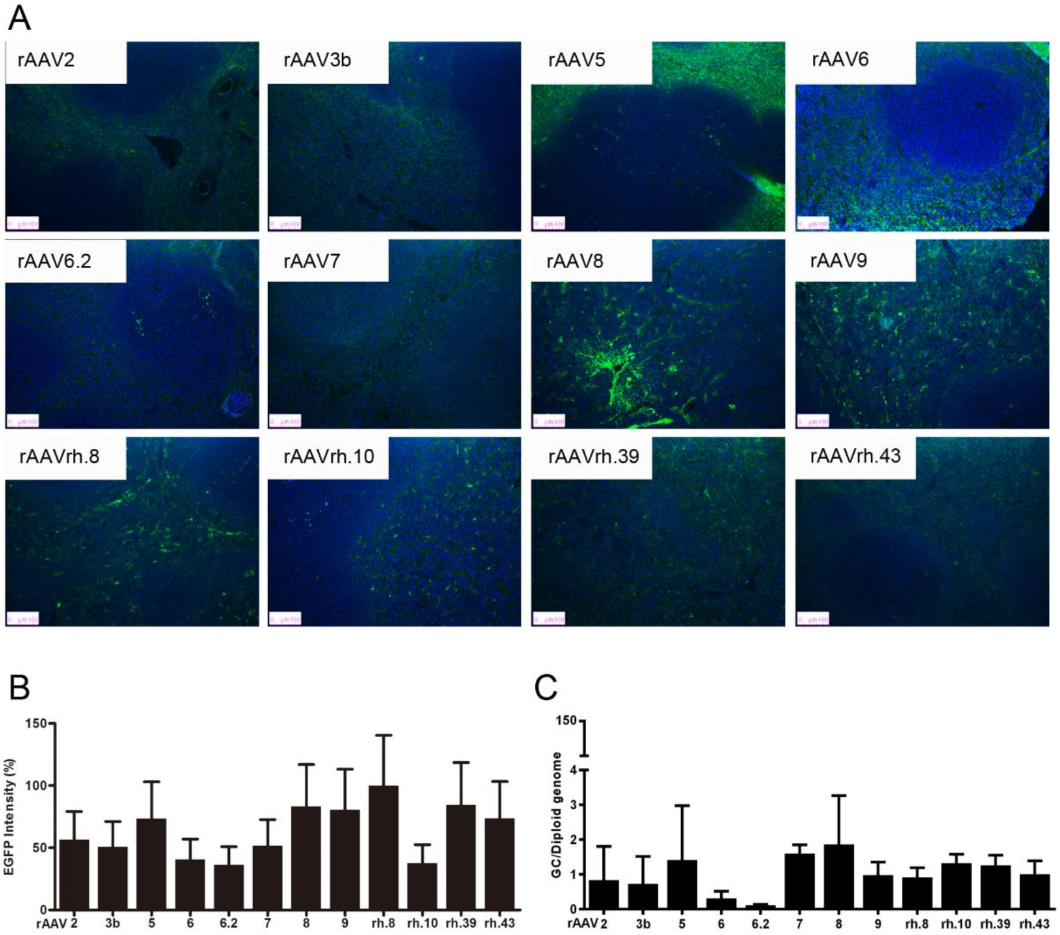

Supplementary Figure 8

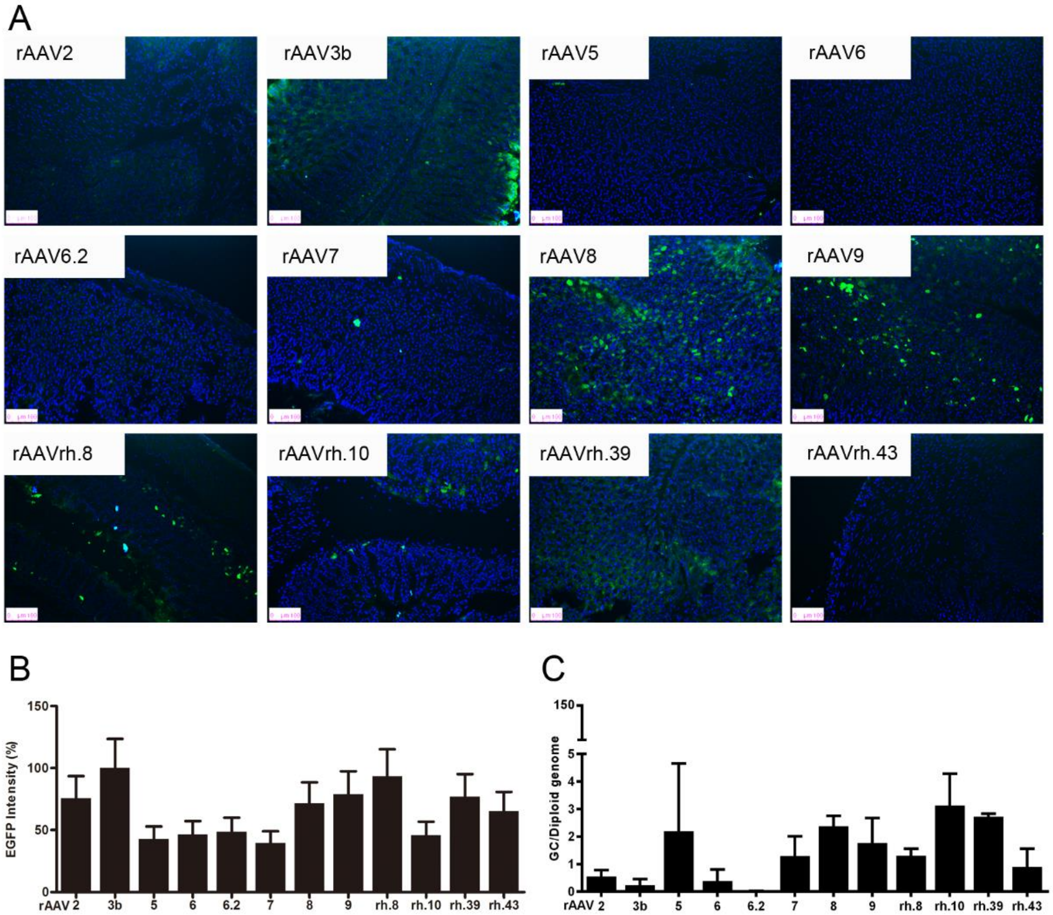

Supplementary Figure 9

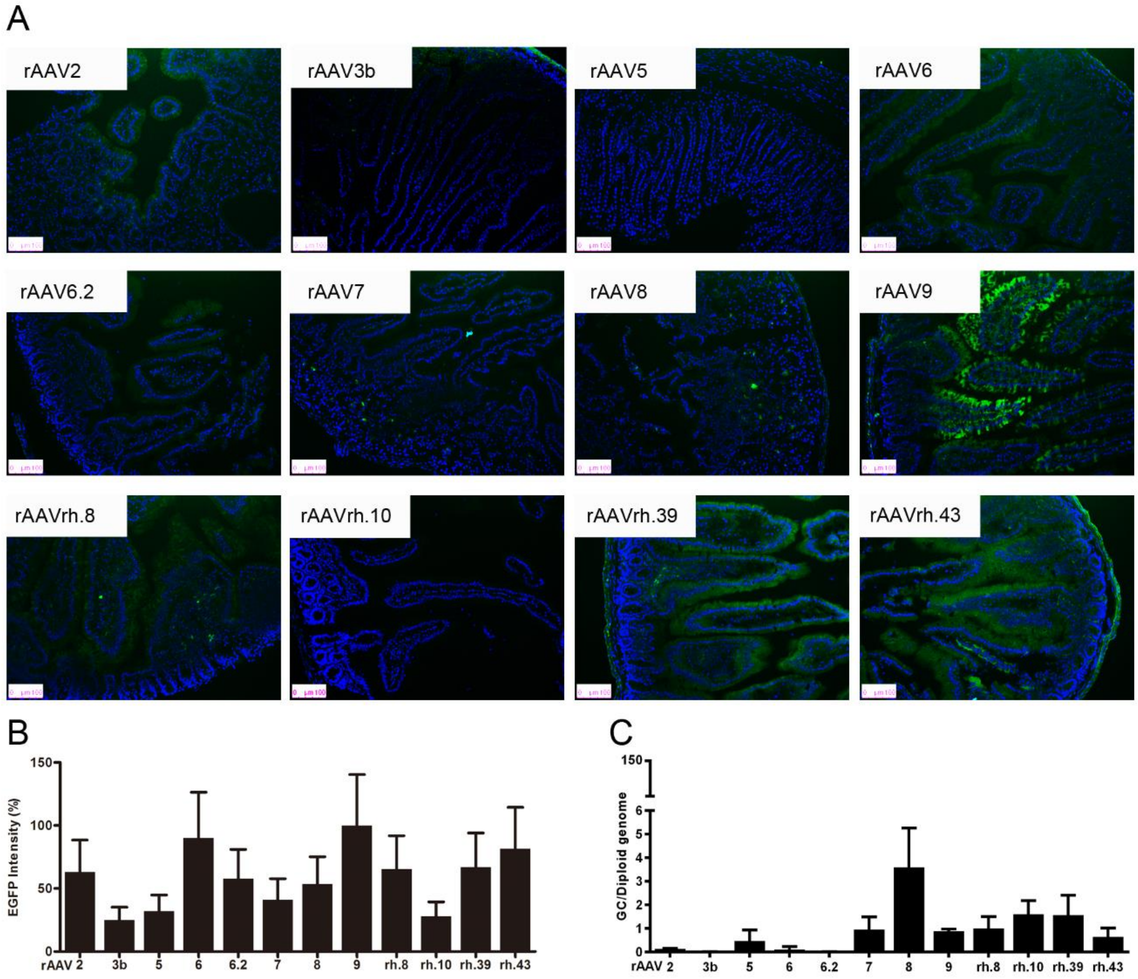

Supplementary Figure 10

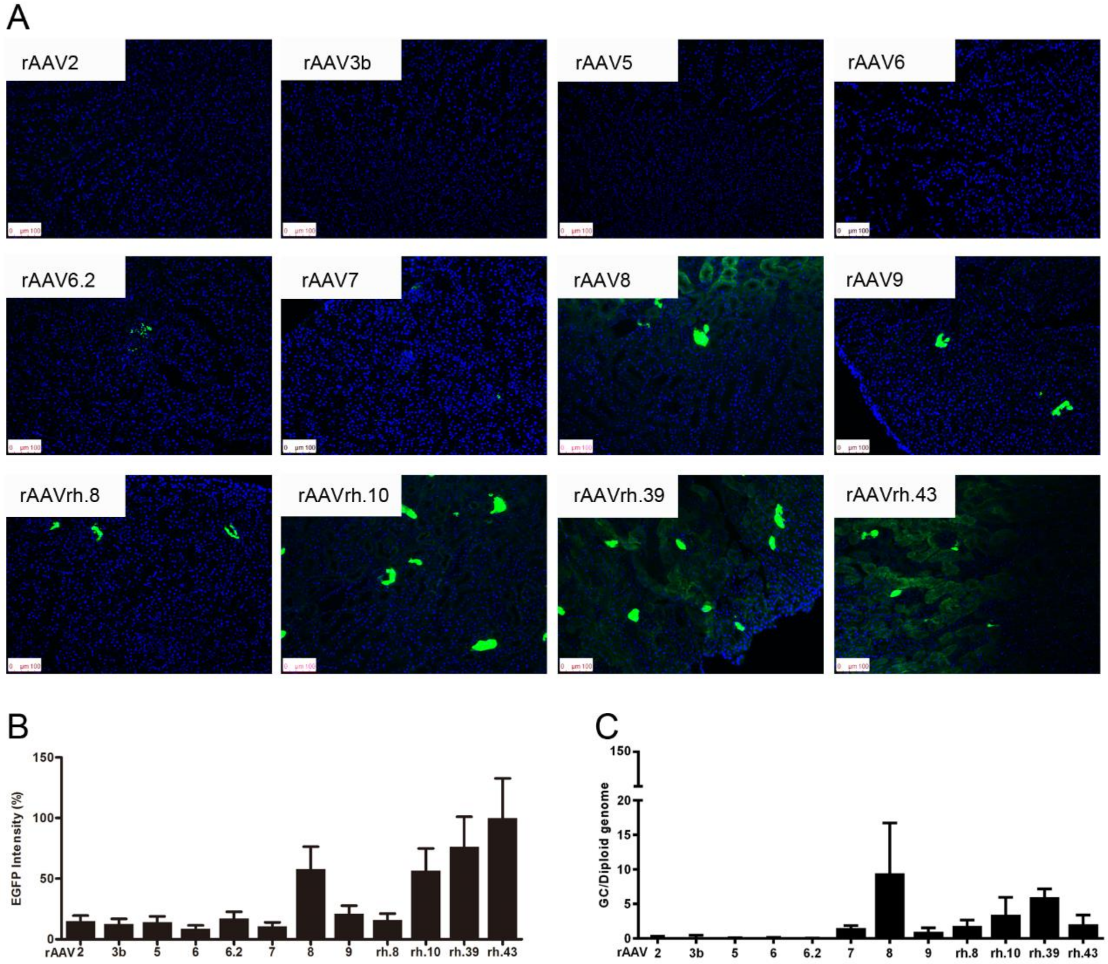

Supplementary Figure 11

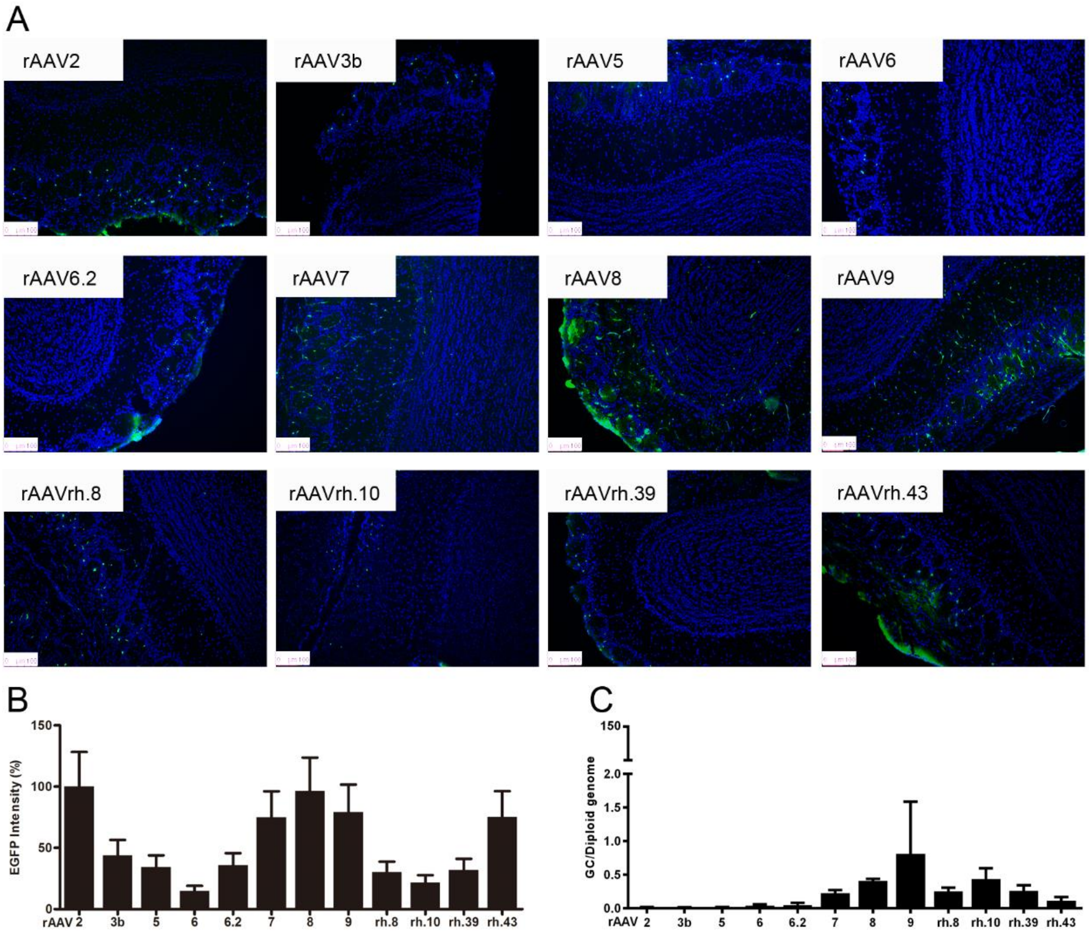

Supplementary Figure 12

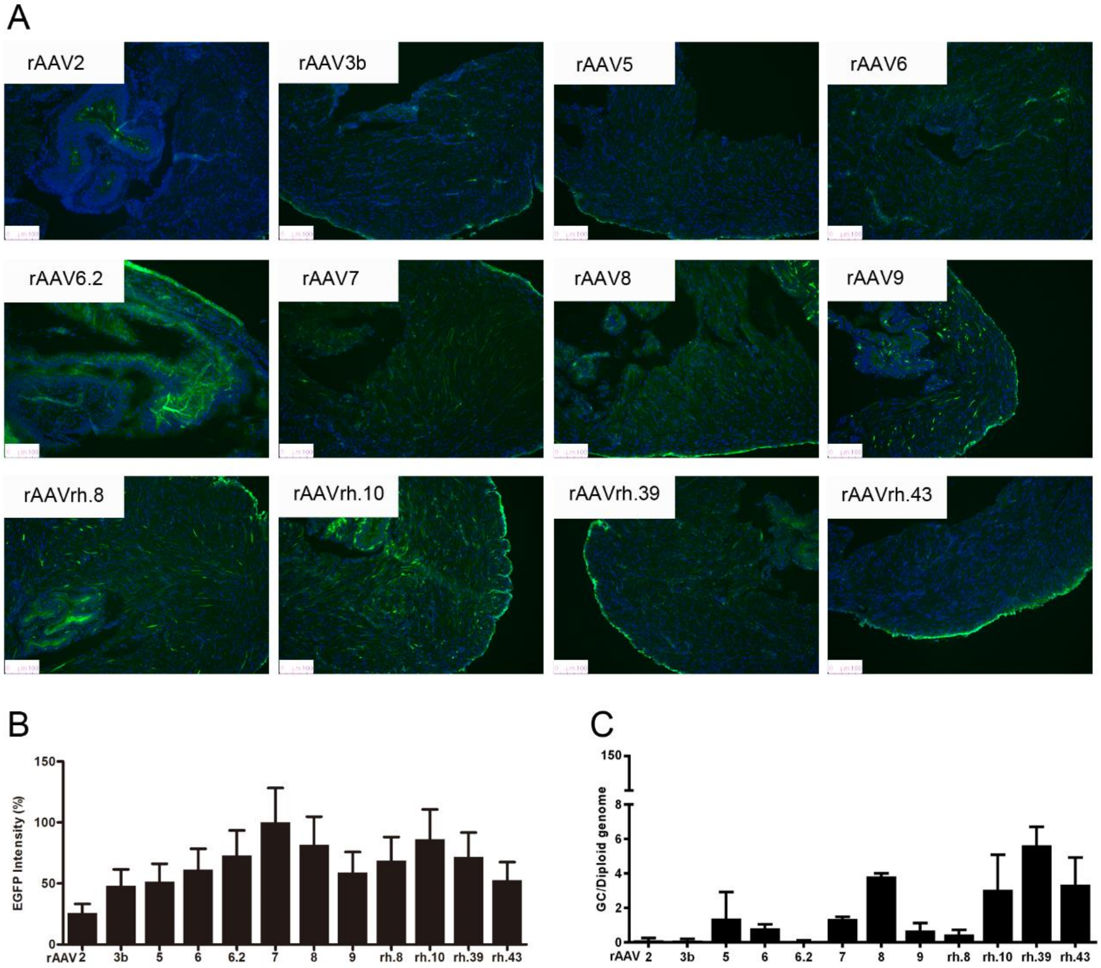

Supplement Figure Legends

Supplement Figure 1. rAAV transduction efficiency in mouse liver. (A) Fluorescence images of liver cryo-sections showing the merge of EGFP native fluorescence (green) and nuclear staining by DAPI (blue) following IP injection. Scale bars represent 100 microns. **(B)** Quantification of transduction efficiency in liver following IP injection with rAAV vectors of different serotypes expressing EGFP. **(C)** Biodistribution of rAAV genomes in liver following IP injection. Data are presented in rAAV genome copies per diploid genome.

Supplement Figure 2. rAAV transduction efficiency in mouse pancreas. (A) Fluorescence images of pancreas cryo-sections showing the merge of EGFP native fluorescence (green) and nuclear staining by DAPI (blue) following IP injection. Scale bars represent 100 microns. **(B)** Quantification of transduction efficiency in pancreas following IP injection with rAAV vectors of different serotypes expressing EGFP. **(C)** Biodistribution of rAAV genomes in pancreas following IP injection. Data are presented in rAAV genome copies per diploid genome.

Supplement Figure 3. rAAV transduction efficiency in mouse skeletal muscle. (A) Fluorescence images of skeletal muscle cryo-sections showing the merge of EGFP native fluorescence (green) and nuclear staining by DAPI (blue) following IP injection. Scale bars represent 100 microns. **(B)** Quantification of transduction efficiency in skeletal muscle following IP injection with rAAV vectors of different serotypes expressing EGFP.

(C) Biodistribution of rAAV genomes in skeletal muscle following IP injection. Data are presented in rAAV genome copies per diploid genome.

Supplement Figure 4. rAAV transduction efficiency in mouse heart. (A) Fluorescence images of heart cryo-sections showing the merge of EGFP native fluorescence (green) and nuclear staining by DAPI (blue) following IP injection. Scale bars represent 100 microns. **(B)** Quantification of transduction efficiency in heart following IP injection with rAAV vectors of different serotypes expressing EGFP. **(C)** Biodistribution of rAAV genomes in heart following IP injection. Data are presented in rAAV genome copies per diploid genome.

Supplement Figure 5. rAAV transduction efficiency in mouse diaphragm. (A) Fluorescence images of diaphragm cryo-sections showing the merge of EGFP native fluorescence (green) and nuclear staining by DAPI (blue) following IP injection. Scale bars represent 100 microns. **(B)** Quantification of transduction efficiency in diaphragm following IP injection with rAAV vectors of different serotypes expressing EGFP. **(C)** Biodistribution of rAAV genomes in diaphragm following IP injection. Data are presented in rAAV genome copies per diploid genome.

Supplement Figure 6. rAAV transduction efficiency in mouse lung. (A) Fluorescence images of lung cryo-sections showing the merge of EGFP native fluorescence (green) and nuclear staining by DAPI (blue) following IP injection. Scale bars represent 100 microns. **(B)** Quantification of transduction efficiency in lung following IP injection with rAAV vectors of different serotypes expressing EGFP. **(C)** Biodistribution of rAAV genomes in lung following IP injection. Data are presented in rAAV genome copies per diploid genome.

Supplement Figure 7. rAAV transduction efficiency in mouse spleen. (A) Fluorescence images of spleen cryo-sections showing the merge of EGFP native fluorescence (green) and nuclear staining by DAPI (blue) following IP injection. Scale bars represent 100 microns. **(B)** Quantification of transduction efficiency in spleen following IP injection with rAAV vectors of different serotypes expressing EGFP. **(C)** Biodistribution of rAAV genomes in spleen following IP injection. Data are presented in rAAV genome copies per diploid genome.

Supplement Figure 8. rAAV transduction efficiency in mouse stomach. (A) Fluorescence images of stomach cryo-sections showing the merge of EGFP native fluorescence (green) and nuclear staining by DAPI (blue) following IP injection. Scale bars represent 100 microns. **(B)** Quantification of transduction efficiency in stomach following IP injection with rAAV vectors of different serotypes expressing EGFP. **(C)**

Biodistribution of rAAV genomes in stomach following IP injection. Data are presented in rAAV genome copies per diploid genome.

Supplement Figure 9. rAAV transduction efficiency in mouse intestine. (A) Fluorescence images of intestine cryo-sections showing the merge of EGFP native fluorescence (green) and nuclear staining by DAPI (blue) following IP injection. Scale bars represent 100 microns. **(B)** Quantification of transduction efficiency in intestine following IP injection with rAAV vectors of different serotypes expressing EGFP. **(C)** Biodistribution of rAAV genomes in intestine following IP injection. Data are presented in rAAV genome copies per diploid genome.

Supplement Figure 10. rAAV transduction efficiency in mouse kidney. (A) Fluorescence images of kidney cryo-sections showing the merge of EGFP native fluorescence (green) and nuclear staining by DAPI (blue) following IP injection. Scale bars represent 100 microns. **(B)** Quantification of transduction efficiency in kidney following IP injection with rAAV vectors of different serotypes expressing EGFP. **(C)** Biodistribution of rAAV genomes in kidney following IP injection. Data are presented in rAAV genome copies per diploid genome.

Supplement Figure 11. rAAV transduction efficiency in mouse brain. (A) Fluorescence images of brain cryo-sections showing the merge of EGFP native fluorescence (green) and nuclear staining by DAPI (blue) following IP injection. Scale

bars represent 100 microns. **(B)** Quantification of transduction efficiency in brain following IP injection with rAAV vectors of different serotypes expressing EGFP. **(C)** Biodistribution of rAAV genomes in brain following IP injection. Data are presented in rAAV genome copies per diploid genome.

Supplement Figure 12. rAAV transduction efficiency in mouse bladder. (A) Fluorescence images of bladder cryo-sections showing the merge of EGFP native fluorescence (green) and nuclear staining by DAPI (blue) following IP injection. Scale bars represent 100 microns. **(B)** Quantification of transduction efficiency in bladder following IP injection with rAAV vectors of different serotypes expressing EGFP. **(C)** Biodistribution of rAAV genomes in bladder following IP injection. Data are presented in rAAV genome copies per diploid genome.

Table 1. Summary table of AAV transduction efficacies

Tissues	Liver		Skeletal muscle		Pancreas		Heart		Diaphragm		Lung		Spleen		Stomach		Intestine		Kidney		Brain		Bladder	
Parameters / rAAV serotypes	⊟ (%)	GC/Diploid genome	⊟ (%)	GC/Diploid genome	⊟ (%)	GC/Diploid genome	⊟ (%)	GC/Diploid genome	⊟ (%)	GC/Diploid genome	⊟ (%)	GC/Diploid genome	⊟ (%)	GC/Diploid genome	⊟ (%)	GC/Diploid genome	⊟ (%)	GC/Diploid genome	⊟ (%)	GC/Diploid genome	⊟ (%)	GC/Diploid genome	⊟ (%)	GC/Diploid genome
**2**	6.7	9.85	34.1	0.13	1.1	0.11	8.2	0.38	49.7	0.99	60.6	0.18	56.4	0.84	75.7	0.56	63.0	0.14	14.8	0.18	100.0	0.02	25.8	0.13
**3b**	1.8	0.37	18.1	0.12	0.8	0.52	10.2	0.04	13.2	0.39	54.7	0.01	50.7	0.73	100.0	0.24	25.0	0.01	12.7	0.22	44.0	0.02	48.0	0.12
**5**	2.5	21.44	29.1	0.24	0.9	3.48	9.4	0.15	8.0	1.67	100.0	0.11	73.4	1.42	42.9	2.19	32.0	0.47	14.2	0.07	34.2	0.02	51.6	1.39
**6**	2.9	2.26	42.5	0.11	0.9	0.82	9.3	0.10	7.2	2.15	69.7	0.10	40.7	0.32	46.4	0.39	90.0	0.11	8.6	0.10	14.8	0.04	61.2	0.83
**6.2**	1.6	0.40	18.3	0.11	0.8	0.02	9.3	0.09	9.0	0.25	55.2	0.01	36.3	0.12	48.6	0.02	57.8	0.01	17.1	0.03	35.6	0.05	72.9	0.06
**7**	68.4	90.57	27.8	2.93	1.3	0.49	15.4	1.97	12.2	1.98	52.8	1.45	51.8	1.60	39.7	1.30	41.2	0.96	10.5	1.54	74.9	0.23	100.0	1.37
**8**	17.0	33.27	45.4	3.76	100.0	9.77	35.8	3.83	96.4	4.68	62.3	1.64	83.3	1.87	71.7	2.38	53.5	3.60	57.7	9.47	96.4	0.41	81.7	3.83
**9**	100.0	44.46	15.5	0.73	27.9	0.95	27.9	1.94	24.4	1.18	83.7	0.56	80.6	0.98	78.9	1.77	100.0	0.89	20.8	1.01	79.2	0.81	59.1	0.70
**rh.8**	9.0	12.59	59.4	0.78	19.7	2.97	31.1	1.83	44.9	1.33	75.4	0.74	100.0	0.92	93.3	1.31	65.5	1.00	16.0	1.85	30.3	0.25	68.6	0.47
**rh.10**	28.4	66.08	100.0	2.32	48.0	10.31	100.0	6.70	100.0	4.28	34.4	1.20	37.4	1.32	46.0	3.13	28.1	1.61	56.5	3.48	21.6	0.44	86.3	3.06
**rh.39**	34.6	110.26	18.6	3.14	54.7	14.25	55.0	3.84	40.3	11.86	51.7	0.93	84.5	1.26	77.1	2.73	67.0	1.57	76.2	6.01	32.0	0.26	71.6	5.63
**rh.43**	55.2	41.65	42.9	0.66	15.4	3.58	12.1	1.07	9.4	0.64	69.3	0.16	73.6	1.01	65.4	0.90	81.5	0.65	100.0	2.06	75.1	0.11	52.7	3.35

Due to the different exposure times of EGFP intensity for different organs entities, the EGFP intensities of different rAAV serotypes are compared within each group of organs (e.g. liver), and not between different organ entities. The highest EGFP intensity of each organ was set to 100% (green color). EI: EGFP intensities; GC: genome copy.

Table 2. Summary Table of Previous Studies

AAV serotypes	Routes	Mice	Authors	Doses
1, 6, 7, 8, 9	intrapericardial/cardiac	Neonatal/adult	Lawrence *et al.*, 2010	2.5E+11/5E+11
1,2,5,6,8,9	Intracranial	8-12-week	Dominik *et al.*, 2013	7.68E+10
1,2,3,4,5,6,7,8,9	tail vein	8-10-week	Carmela *et al.*, 2008	1.00E+11
1,2,5	intracerebral	adult	Corinna *et al.*, 2004	~2E+10
1,2,5,6,7,8	intravenous/intramuscular	Neonatal/adult	Wang *et al.*, 2005	2E+11/2E+12/4E+10
2,3b,5,6,6.2,7,8,9,rh.8, rh.10,rh.39,rh.43	intraperitoneal	8-week	Ai *et al.* Current Study	1.00E+12
